# Ischemia-induced endogenous Nrf2/HO-1 axis activation modulates microglial polarization and restrains ischemic brain injury

**DOI:** 10.3389/fimmu.2024.1440592

**Published:** 2024-10-14

**Authors:** Ping-Chang Kuo, Wen-Tsan Weng, Barbara A. Scofield, Hallel C. Paraiso, I-Chen Ivorine Yu, Jui-Hung Jimmy Yen

**Affiliations:** ^1^ Department of Microbiology and Immunology, Indiana University School of Medicine, Fort Wayne, IN, United States; ^2^ Department of Anatomy, Cell Biology and Physiology, Indiana University School of Medicine, Fort Wayne, IN, United States

**Keywords:** ischemic stroke, Nrf2/HO-1 axis, microglia, diabetic stroke, neuroinflammation

## Abstract

Cerebral ischemic stroke accounts for more than 80% of all stroke cases. During cerebral ischemia, reactive oxygen species produced in the ischemic brain induce oxidative stress and inflammatory responses. Nrf2 is a transcription factor responsible for regulating cellular redox balance through the induction of protective antioxidant and phase II detoxification responses. Although the induction of endogenous Nrf2/HO-1 axis activation has been observed in the ischemic brain, whether ischemia-induced endogenous Nrf2/HO-1 axis activation plays a role in modulating microglia (MG) phenotypes and restraining ischemic brain injury is not characterized and requires further exploration. To investigate that, we generated mice with Nrf2 knockdown specifically in MG to rigorously assess the role of endogenous Nrf2 activation in ischemic brain injury after stroke. Our results showed that MG-specific Nrf2 knockdown exacerbated ischemic brain injury after stroke. We found that Nrf2 knockdown altered MG phenotypes after stroke, in which increased frequency of inflammatory MG and decreased frequency of anti-inflammatory MG were detected in the ischemic brain. Moreover, we identified attenuated Nrf2/HO-1 axis activation led to increased CD68/IL-1β and suppressed CD206 expression in MG, resulting in aggravated inflammatory MG in MG-specific Nrf2 knockdown mice after stroke. Intriguingly, using type II diabetic preclinical models, we revealed that diabetic mice exhibited attenuated Nrf2/HO-1 axis activation in MG and exacerbated ischemic brain injury after stroke that phenocopy mice with MG-specific Nrf2 knockdown. Finally, the induction of exogenous Nrf2/HO-1 axis activation in MG through pharmacological approaches ameliorated ischemic brain injury in diabetic mice. In conclusion, our findings provide cellular and molecular insights demonstrating ischemia-induced endogenous Nrf2/HO-1 axis activation modulates MG phenotypes and restrains ischemic brain injury. These results further strengthen the therapeutic potential of targeting Nrf2/HO-1 axis in MG for the treatment of ischemic stroke and diabetic stroke.

## Introduction

Stroke is a leading cause of death and a major cause of disability worldwide. Approximately 87% of all stroke cases are ischemic stroke (1, 2). Currently, the only approved pharmacological treatment for acute ischemic stroke is intravenous (i.v.) thrombolysis with recombinant tissue plasminogen activator (tPA), aiming to reestablish cerebral blood flow (3). However, the treatment of tPA has significant clinical limitations and does not modulate the complex events taking place after the onset of ischemic stroke, including oxidative stress and neuroinflammation (4, 5). In addition, reperfusion increases the generation of reactive oxygen species and promotes the infiltration of peripheral inflammatory immune cells into the ischemic brain, further aggravating neuroinflammation and exacerbating ischemic brain injury (6).

The transcription factor nuclear factor E2-related factor 2 (Nrf2) is a major regulator of cellular defense mechanism against endogenous and exogenous oxidative stresses. The induction of Nrf2 pathway has been observed in various ischemic injuries (7–10). Under homeostatic conditions, Nrf2 is associated with kelch-like ECH-associated protein 1 (Keap1) and the adapter protein of E3 ubiquitin ligase and is rapidly degraded through ubiquitination (11, 12). However, under stress conditions, Nrf2 is dissociated from the Keap1 complex and then translocated into the nucleus. In the nucleus, Nrf2 heterodimerizes with small Maf proteins and binds to antioxidant response elements, leading to the induction of a variety of phase II antioxidant genes, including NAD(P)H qui-none oxidoreductase 1 (NQO1), heme-oxygenase (HO-1), and glutamate-cysteine ligase catalytic subunit (GCLC) (13). In addition to its antioxidant effects, the induction of Nrf2/HO-1 axis has been shown to exert anti-inflammatory effects through regulating NLRP3 inflammasome and NFκB pathway activation (14–17).

Studies have demonstrated that compounds, inducing exogenous Nrf2 activation in the central nerve system (CNS), offer protection against neuroinflammation, including ischemic stroke and multiple sclerosis (18–21). In addition, the induction of endogenous Nrf2/HO-1 axis is observed in the ischemic brain (22–24). However, whether the induction of endogenous Nrf2/HO-1 axis specifically in microglia (MG) plays an essential role in modulating MG phenotypes and restraining ischemic brain injury following stroke is under characterized and requires further investigation. In this study, we generated mice with MG-specific Nrf2 knockdown to assess the effect of endogenous Nrf2/HO-1 axis activation in MG on modulating MG phenotypes and ischemic brain injury after stroke. Furthermore, we employed type II diabetic preclinical models to elucidate the essential role of endogenous Nrf2/HO-1 axis activation in MG in restraining ischemic brain injury after stroke. Here, we showed that MG-specific Nrf2 knockdown led to exacerbated ischemic brain injury after stroke. At the cellular level, we demonstrated that MG-specific Nrf2 knockdown altered MG phenotypes, in which increased inflammatory MG and decreased anti-inflammatory MG were observed in the ischemic brain. At the molecular level, we observed that diminished Nrf2/HO-1 axis activation in MG resulted in increased CD68/IL-1β and suppressed CD206 expression in MG, leading to the elevated inflammatory MG in MG-specific Nrf2 knockdown stroke mice. Intriguingly, we found that diabetic stroke mice exhibited attenuated Nrf2/HO-1 axis activation in MG and exacerbated ischemic brain injury, phenocopying MG-specific Nrf2 knockdown stroke mice. Importantly, the pharmacological induction of exogenous Nrf2/HO-1 axis activation in MG ameliorated diabetes-exacerbated brain injury after ischemic stroke. Our findings provide molecular and cellular insights into the protective effects of endogenous Nrf2/HO-1 axis activation in MG following ischemic stroke and further strengthen the therapeutic potential of targeting Nrf2/HO-1 axis activation in MG for the treatment of ischemic stroke.

## Materials and methods

### Mice

Wild-type (WT) C57BL/6, *Cx3cr1^CreERT2/CreERT2^*, *Nrf2^flox/flox^*, *Nrf2^-/-^*, and *Lepr^db/db^* mice were purchased from the Jackson Laboratory (Bar Harbor, ME) and bred in our animal facility. All animal experimental procedures conducted in this study were approved by the Purdue Animal Care and Use Committee and performed in strict compliance with the National Institutes of Health Guide for the Care and Use of Laboratory Animals. To achieve MG-specific Nrf2 knockdown, *Nrf2^fl/fl^-Cx3cr1^CreERT2/+^* mice were generated and subjected to i.p. administration of 75 mg/kg tamoxifen (TAM) for 5 consecutive days. Following TAM treatment, *Nrf2^fl/fl^-Cx3cr1^CreERT2/+^* mice were housed for additional 8 weeks, allowing peripheral monocytes/macrophages to be replenished. Control *Cx3cr1^CreERT2/+^* mice were subjected to the same procedure of TAM treatment. All mice were housed and bred with controlled humidity, temperature, and a 12 h:12 h light-dark cycle in the animal facility with food and water available ad libitum.

### Reagents

Triphenyltetrazolium chloride (TTC) and trichloroacetic acid (TCA) were purchased from Alfa Aesar (Tewksbury, MA). Evans blue and dimethyl itaconate (DMI) were purchased from Sigma-Aldrich (St. Louis, MO). Flow cytometry antibodies of Alexa Fluor 488 anti-CD45 (Clone: 30-F11), APC anti-CD11b (Clone: M1/70), PE anti-Nrf2 (Clone: W19086B), PE/Cy7 anti-CD68 (Clone: FA-11), and PE/Cy7 anti-CD206 (Clone: C068C2) and western blot antibodies of MMP3 (Clone: M4405F10) and MMP9 (Clone: L51/82) as well as the True-Nuclear™ transcription factor buffer set were purchased from BioLegend (San Diego, CA). APC anti-IL-1β (Clone: NJTEN3) and Alexa Fluor 546 anti-rabbit IgG antibodies and Pierce™ BCA Protein Assay Kit were purchased from Thermo Fisher Scientific (Waltham, MA). Anti-HO-1 antibody was purchased from Proteintech (Rosemont, IL).

### Middle cerebral artery occlusion model

Cerebral ischemia was induced in adult male and female mice (3-5 months old) as previously described (25). Briefly, mice under anesthesia were subjected to intraluminal occlusion of the right middle cerebral artery through the insertion of a silicone-coated nylon monofilament (Doccol Corp, Sharon, MA). Cerebral blood flow (CBF) was measured via laser Doppler flowmetry. Male mice were subjected to a 40 min occlusion, and female mice were subjected to a 1 h occlusion based on previously established conditions (18). Sham controls were generated by the same surgical procedure but without inserting the monofilament. The animal’s body temperature was maintained at ~37°C throughout the surgery by a warming lamp and heating pad. After surgery, mice were placed in a recovery cage, where the temperature was maintained at 37 °C, for 1 h to recover from anesthesia. Mice that had a total CBF reduction of less than 80% during surgery were excluded from the study because they did not meet the surgical standard. Mice subjected to Evans blue extravasation assay were occluded for 3 h based on previous established criteria (21, 26, 27). *Lepr^db/db^* mice were subjected to a reduced occlusion time of 30 min because they developed severe brain injury compared to WT mice after ischemic stroke. Hyperglycemia is observed in acute ischemic stroke and highly correlated with the infarct volume of ischemic brain tissue (28, 29). To ensure rigor, the blood glucose levels of *Lepr^db/db^* mice were measured during MCAO, and *Lepr^db/db^* MCAO mice with blood glucose levels less than 400 mg/dL during occlusion were excluded from the study because these mice did not consistently develop enlarged infarct volumes after ischemic stroke. For the treatment, *Lepr^db/db^* MCAO mice were randomly assigned to receive i.p. vehicle (PBS) or DMI (500 mg/kg) at the indicated time. The dose of 500 mg/kg DMI used in this study was based on our previous study in which we observed DMI induced HO-1 expression in MG and attenuated infarct volume in C57/B6 mice subjected to MCAO (27). To enhance rigor, in addition to sham controls the contralateral hemispheres of ischemic brains were also included in all studies as additional controls. The investigators who performed the experiments were blinded to the animal groups.

### Infarct volume and edema measurements

At the indicated times, mice were anesthetized deeply and perfused with PBS, and the ischemic brains were harvested and subjected to 2 mm coronal slicing with a rodent brain matrix. Brain sections were then stained with 1% TTC followed by scanning, and the infarct volume was calculated by using ImageJ as previously described (30). The cerebral edema ratio was calculated by comparing the cerebral volume of the ipsilateral hemisphere (Vi) to the cerebral volume of the contralateral hemisphere (Vc) using the equation of Vi/Vc.

### Mononuclear cell isolation and flow cytometry analysis

Mice were anesthetized deeply and transcardially perfused with PBS at the indicated time points. The brains were harvested, and the meninges, olfactory bulb, and cerebellum were removed. The forebrains were homogenized with 1X Hanks’ balanced salt solution buffer and then filtered through a 70 μm nylon cell strainer. Cells were centrifuged, resuspended in 30% Percoll, and underlayered with 70% Percoll. After centrifugation, the mononuclear cells were isolated from the interface between 30% and 70% Percoll. For intracellular staining, the isolated cells were stained with antibodies against CD45 and CD11b followed by fixation and permeabilization. After washing, the cells were stained with HO-1, CD68, or CD206 antibodies, followed by flow cytometry analysis. For intranuclear staining, the isolated cells were stained with antibodies against CD45 and CD11b followed by fixation and permeabilization using the True-Nuclear™ transcription factor buffer set. After washing, the cells were stained with Nrf2 antibody followed by flow cytometry analysis. For intracellular cytokine staining, the isolated cells were cultured *ex vivo* in the presence of GolgiPlug for 4.5 h. Cells were then collected and stained with antibodies against CD45 and CD11b followed by fixation and permeabilization. After washing, the cells were stained with IL-1β antibodies followed by flow cytometry analysis. The population of MG was determined based on their intermediate expression of CD45 (CD45^int^) and positive expression of CD11b (CD11b^+^). The gating strategy is presented in **Supplementary Figure 1**.

### Evans blue extravasation assay

Mice were i.v. administered 4 ml/kg 2% (w/v) Evans blue dye/0.9% saline solution. One hour after injection, mice were anesthetized deeply and perfused with PBS to remove intravascular Evans blue. The ischemic brains were harvested, sliced, and subjected to scanning to obtain images of Evans blue extravasation. In addition, the hemispheres of brain sections were separated and weighed followed by homogenization with 50% TCA solution. After centrifugation, the supernatants were collected and diluted with 95% ethanol to a ratio of 1:3. The amount of extravascular Evans blue in the supernatants was determined by measuring the fluorescence with excitation at 540/25 nm and emission at 645/40 nm using a BioTek Synergy HT microplate reader.

### Western blot analysis

RIPA buffer [50 mM Tris-HCl (pH 8.0), 150 mM NaCl, 1% NP-40, 0.5% sodium deoxycholate, 1 mM PMSF, and 1X protease inhibitor cocktail with 0.3% SDS] was used to prepare protein samples. The Pierce™ BCA Protein Assay Kit was used to measure the protein concentrations of the samples. SDS‒PAGE (10%) was prepared to conduct protein electrophoresis. After transferring to polyvinylidene difluoride membranes, the membrane blots were reacted with primary MMP3 and MMP9 antibodies followed by secondary antibodies to detect the protein signals using Immobilon Western Chemiluminescent HRP Substrate.

### Statistical analysis

All results are given as the mean ± SEM in this study. Sample sizes were determined by power calculations based on our previous studies (26, 27, 30). The normal distribution of the data was confirmed by the Shapiro‒Wilk normality test. For samples passing normality test, comparisons between two groups were performed by unpaired *t* test and comparisons among multiple groups were performed by one-way ANOVA (one variable) or two-way ANOVA (two variables) followed by Tukey’s *post hoc* test. For samples that did not pass normality test, comparisons between two groups and one variable were performed by Mann-Whitney *U* test. Statistical analyses were performed by using GraphPad Prism 10 software. Statistical significance was determined as *p<0.05*.

## Results

### Temporal expression of HO-1, CD206, and CD68 in MG after ischemic stroke injury

**Figure 1:**
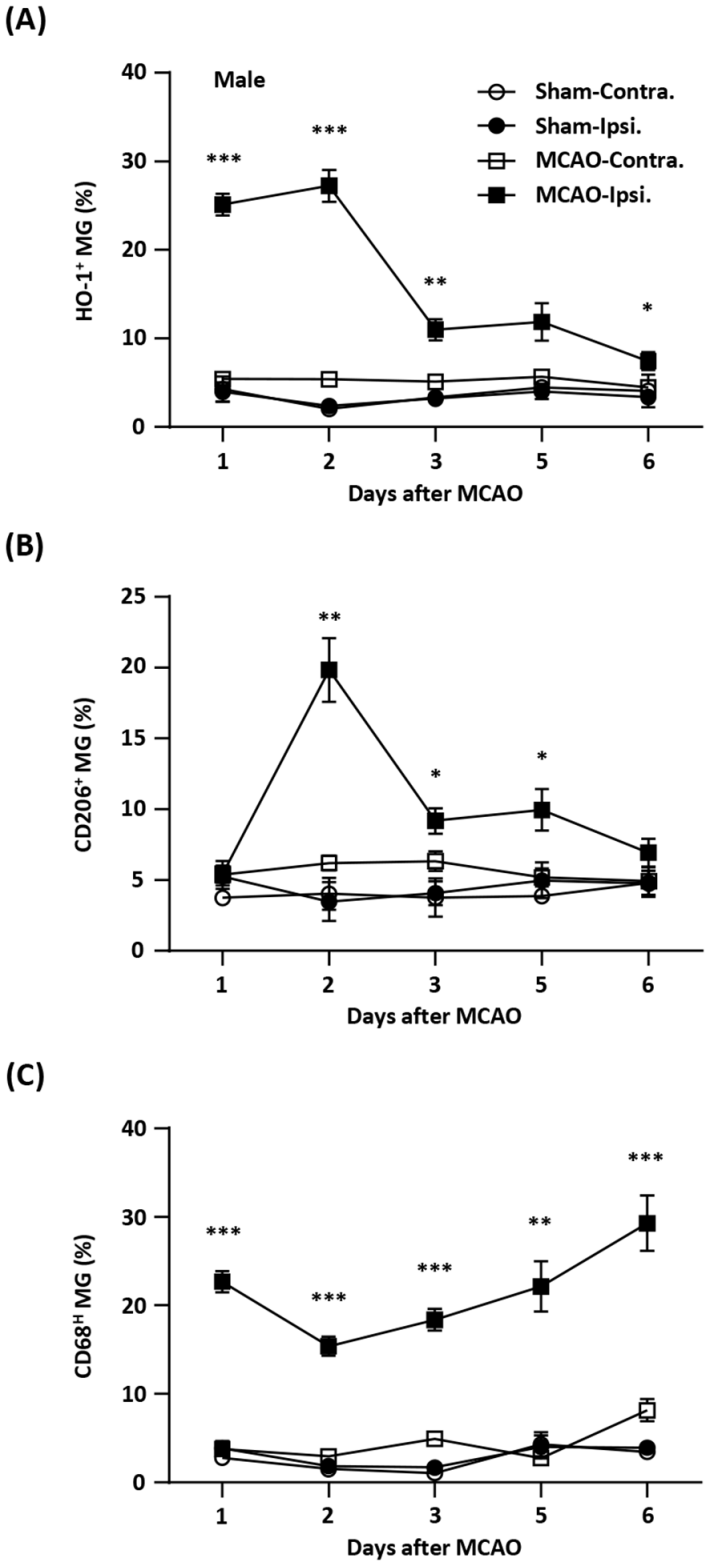
Temporal expression of HO-1, CD206, and CD68 in MG after ischemic stroke. Male C57BL/6 mice were subjected to sham (n=3/per time point of day 1-6) or 40 min MCAO (n=10/per time point of day 1-5; n=6/day 6). On day 1, 2, 3, 5, and 6 post-injury, mice were sacrificed, and the ischemic brains were harvested. The contralateral (Contra.) and ipsilateral (Ipsi.) hemispheres of sham and MCAO mice were subjected to mononuclear cell isolation. The isolated cells were stained with antibodies against CD45 and CD11b followed by intracellular staining with HO-1, CD206, or CD68 to assess CD45^int^CD11b^+^ MG positive for **(A)** HO-1, **(B)** CD206, or **(C)** CD68 expression, respectively. Statistical analysis was comparing the ipsilateral hemisphere of MCAO mice to that of sham controls. *p<0.05; **p<0.01; ***p<0.001 by unpaired *t* test or Mann-Whitney *U* test.

Induction of exogenous Nrf2/HO-1 axis in MG has been shown to modulate neuroinflammation (16–22, 31–34). However, the effect of endogenous Nrf2/HO-1 axis activation specifically in MG on ischemia-induced neuroinflammation is under characterized. To determine whether the induction of endogenous Nrf2/HO-1 axis affects MG phenotypes after ischemic stroke, we conducted a time course study and assessed MG phenotypes in the ischemic brain. Mice were subjected to sham or MCAO and sacrificed on days 1, 2, 3, 5, and 6 post-injury. The ischemic brains were harvested to determine HO-1, CD206, and CD68 expression in MG. The expression of CD206 or CD68 in MG was defined as an anti-inflammatory or inflammatory phenotype, respectively, based on previous studies (26, 35–37). Our results showed that ischemia significantly increased frequency of HO-1-expressing MG in the ipsilateral hemisphere in MCAO mice compared to the contralateral hemisphere in MCAO mice and both hemispheres in sham controls on day 1 and day 2 post-injury (**Figure 1A**). However, the frequency of HO-1-expressing MG was dramatically reduced on day 3 post-injury and further decreased on day 6 post-injury (**Figure 1A**). We then determined the frequency of CD206-expressing MG in MCAO mice. Although there was no induction of CD206-expressing MG in the ipsilateral hemisphere of MCAO mice compared to the contralateral hemisphere of MCAO mice and the both hemispheres of sham controls on day 1 post-injury, the frequency of CD206-expressing MG was profoundly elevated on day 2 post-injury (**Figure 1B**). Notably, the frequency of CD206-expressing MG was then declined on day 3 through day 6 post-injury (**Figure 1B**). Finally, the frequency of CD68-expressing MG in the ischemic brain was assessed. Intriguingly, we found a reciprocal correlation between CD68- and CD206-expressing MG in the ischemic brain. The frequency of CD68-expressing MG was increased on day 1 post-injury but decreased on day 2 post-injury. However, the frequency of CD68-expressing MG gradually increased on day 3 through day 6 post-injury (**Figure 1C**). Collectively, our findings demonstrate a positive correlation between HO-1- and CD206- expressing MG but a negative correlation between HO-1- and CD68-expressing MG in the ischemic brain. These results imply the potential interplay among HO-1, CD206, and CD68 expression in MG in the ischemic bran that may exert effects on modulating MG phenotypes following ischemic stroke.

### Ischemia-induced HO-1, CD206, and CD68 expression in MG is comparable between female and male MCAO mice

**Figure 2:**
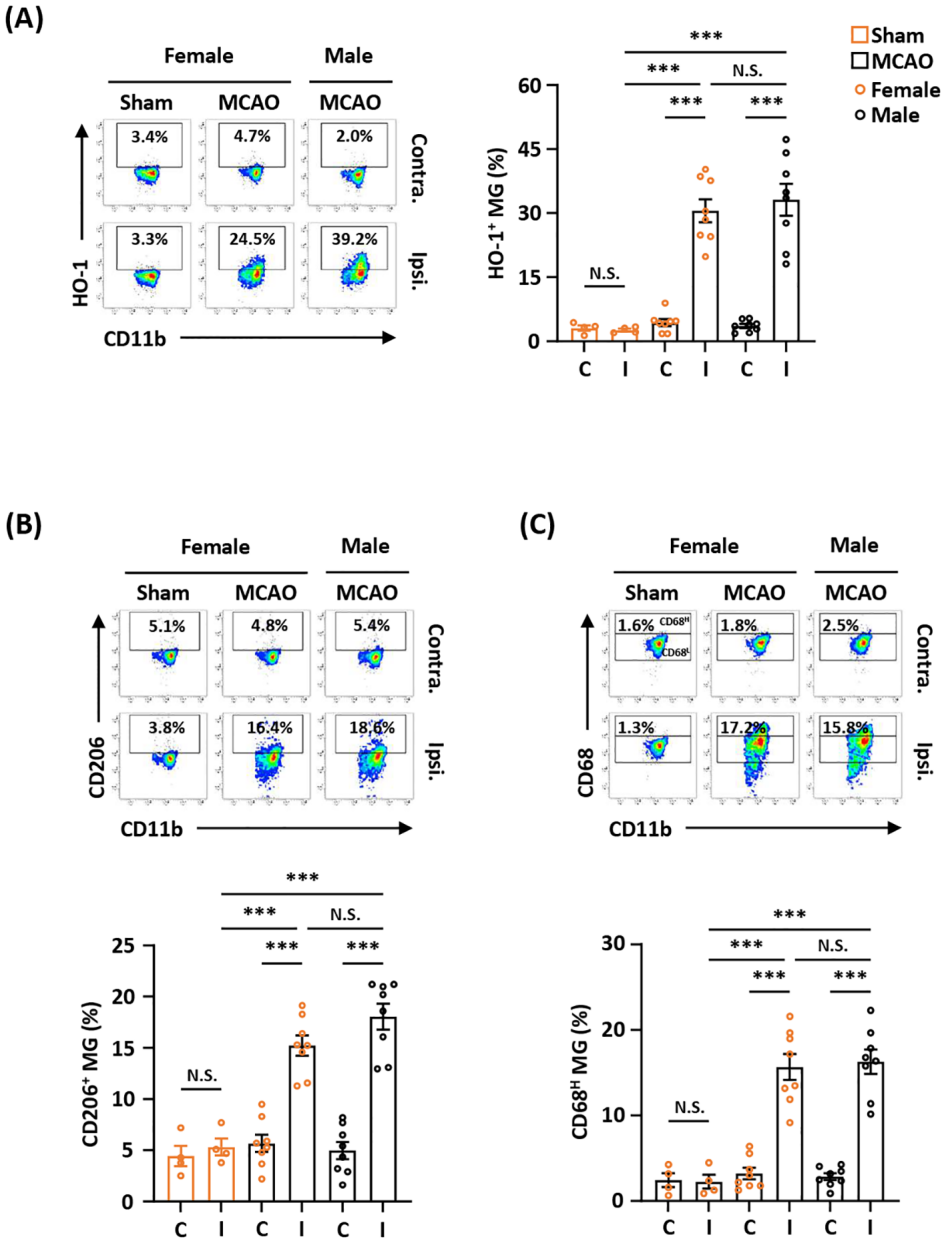
Ischemia-induced HO-1, CD206, and CD68 expression in MG is comparable between female and male MCAO mice. Female C57BL/6 mice were subjected to sham (n=4/group) or MCAO (n=8/group). Male C57BL/6 mice were also subjected to MCAO (n=8/group). On day 2 post-injury, the contralateral (Contra.; C) and ipsilateral (Ipsi.; I) hemispheres of sham and MCAO mice were harvested, followed by mononuclear cell isolation. The isolated cells were stained with CD45 and CD11b in the presence of HO-1, CD206, or CD68 to assess CD45^int^CD11b^+^ MG positive for **(A)** HO-1, **(B)** CD206, or **(C)** CD68 expression, respectively. The gating of CD68 low (CD68^L^) was based on the basal level of CD68 expression in MG in sham controls compared to isotype, and the expression level of CD68 higher than CD68^L^ was then determined as CD68 high (CD68^H^). ***p<0.001; N.S.: no significant difference by two-way ANOVA.

Previous studies show that there is a sex difference in MG (38, 39). To determine whether sex affects ischemia-induced HO-1, CD206, and CD68 expression in MG after ischemic brain injury, female and male mice were subjected to MCAO, and the ischemic brains were then harvested to assess the expression of HO-1, CD206, and CD68 in MG. Our results showed that the frequency of HO-1-expressing MG was comparable in female and male MCAO mice (**Figure 2A**). In addition, the frequency of CD206- and CD68-expressing MG was also comparable in female and male MCAO mice (**Figures 2B, C**). Altogether, our results demonstrate that ischemic stroke induces comparable levels of HO-1, CD206, and CD68 expression in MG in the ischemic brains of female and male MCAO mice, suggesting sex does not affect ischemia-induced MG phenotypes after ischemic stroke.

### MG-specific Nrf2 knockdown exacerbates ischemic brain injury after stroke

**Figure 3:**
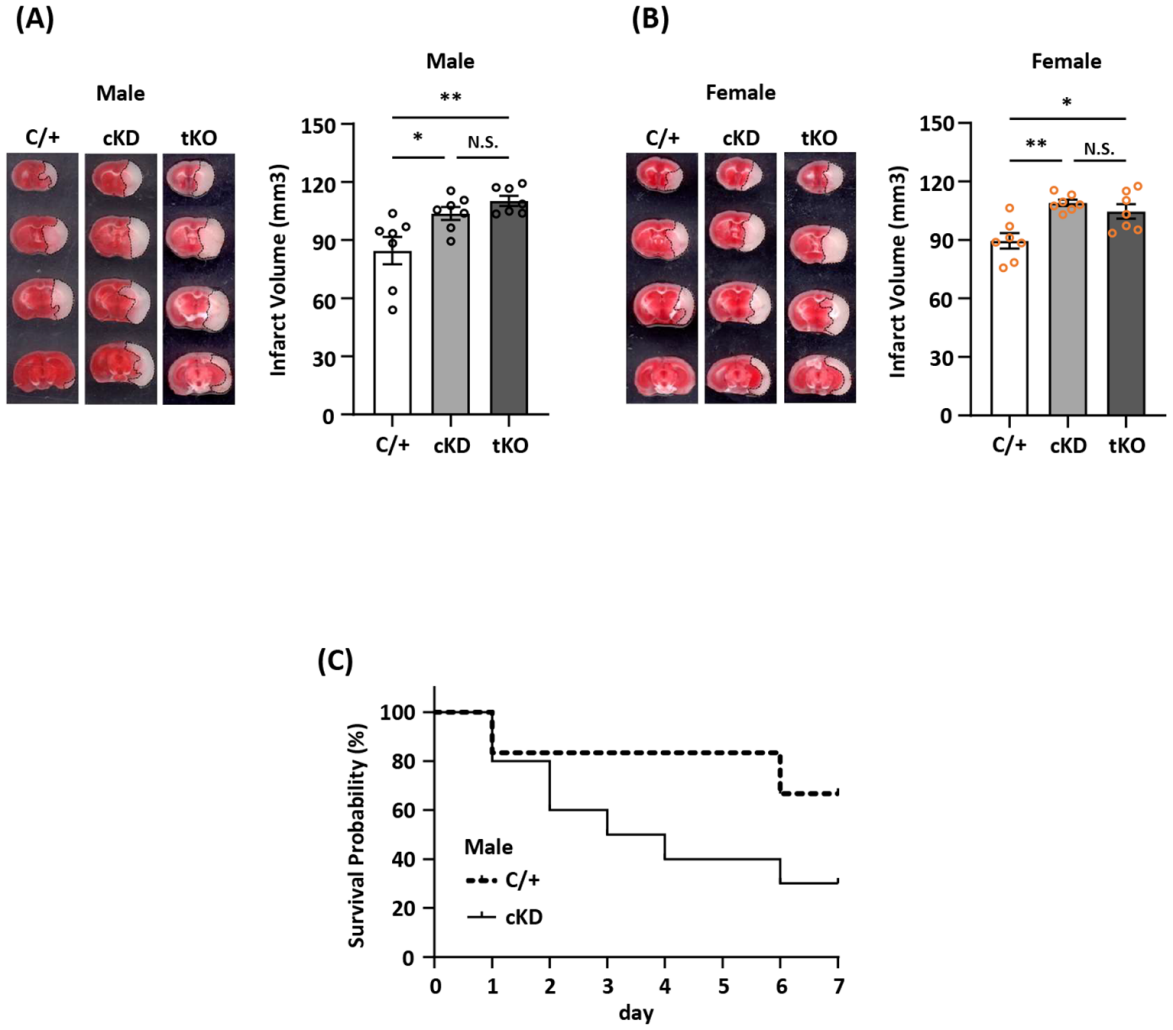
MG-specific Nrf2 knockdown exacerbates ischemic brain injury after stroke. **(A)** Male and **(B)** female *Cx3cr1^CreERT2/+^* (C/+)*, Nrf2^fl/fl^-Cx3cr1^CreERT2/+^* (conditional knockdown; cKD), and *Nrf2^-/-^* (total knockout; tKO) mice were subjected to MCAO (n=7/group). On day 2 post-injury, the ischemic brains were harvested and subjected to TTC staining. One representative TTC-stained brain sample from each group is shown, and the infarct volumes were measured. *p<0.05; **p<0.01; N.S.: no significant difference by one-way ANOVA. **(C)** Male C/+ (n=6) and cKD (n=10) mice were subjected to MCAO and monitored to assess the survival rate from day 0 to day 7 post-injury.

The induction of Nrf2/HO-1 pathway has been shown to exert anti-inflammatory and antioxidant effects in peripheral and CNS diseases. However, whether ischemia-induced endogenous Nrf2/HO-1 axis activation in MG plays a role in modulating ischemic brain injury is underexplored. To investigate that, we generated mice with MG-specific Nrf2 knockdown and subjected male and female *Nrf2^fl/fl^-Cx3cr1^CreERT2/^* ^+^ mice to MCAO. At day 2 post-injury, the ischemic brains were harvested to determine infarct volumes. In addition, male and female *Cx3cr1^CreERT2/^* ^+^ control mice and total Nrf2 knockout (*Nrf2^-/-^*) mice were also subjected to MCAO to assess the level of brain injury. Our results showed that male *Nrf2^fl/fl^-Cx3cr1^CreERT2/^* ^+^ MCAO mice displayed significantly larger infarct volumes than control male *Cx3cr1^CreERT2/^* ^+^ MCAO mice (**Figure 3A**). Notably, we observed that male *Nrf2^fl/fl^-Cx3cr1^CreERT2/^* ^+^ and *Nrf2^-/-^* MCAO mice developed a comparable level of infarct volumes, suggesting MG may represent the main Nrf2-expressing cells in the CNS to modulate ischemic brain injury after stroke (**Figure 3A**). Furthermore, we assessed the effect of MG-specific Nrf2 knockdown in female MCAO mice to evaluate whether there is a sex difference. Consistently, our results showed that female *Nrf2^fl/fl^-Cx3cr1^CreERT2/^* ^+^ MCAO mice developed larger infarct volumes than control female *Cx3cr1^CreERT2/+^* MCAO mice (**Figure 3B**). We also observed a comparable level of infarct volumes between female *Nrf2^fl/fl^-Cx3cr1^CreERT2/^* ^+^ and *Nrf2^-/-^* MCAO mice (**Figure 3B**). These results suggest there is no sex difference in ischemic brain injury under the condition of MG-specific Nrf2 knockdown. Finally, we assessed long-term survival in male *Nrf2^fl/fl^-Cx3cr1^CreERT2/^* ^+^ and *Cx3cr1^CreERT2/^* ^+^ MCAO mice. Our results showed that male *Nrf2^fl/fl^-Cx3cr1^CreERT2/^* ^+^ MCAO mice exhibited reduced survival compared to male *Cx3cr1^CreERT2/^* ^+^ MCAO mice (**Figure 3C**). Altogether, our results demonstrate that MG-specific Nrf2 knockdown exacerbates brain injury and diminishes survival after ischemic stroke, suggesting the induction of endogenous Nrf2 activation in MG plays an essential role in restraining ischemic brain injury after stroke.

### MG-specific Nrf2 knockdown results in attenuated HO-1 expression in the ischemic brain after stroke

**Figure 4:**
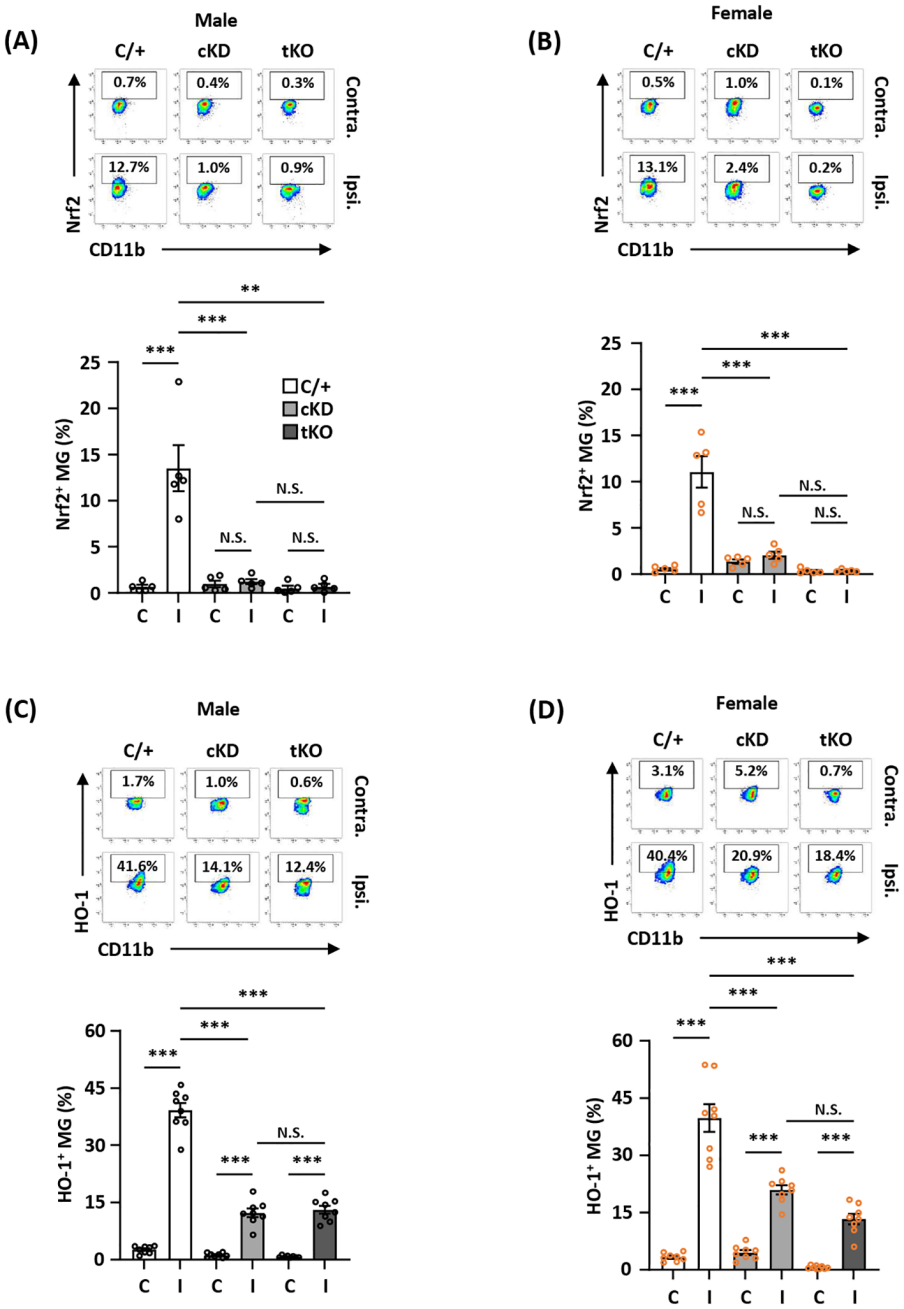
MG-specific Nrf2 knockdown results in attenuated HO-1 expression in the ischemic brain after stroke. Male and female *Cx3cr1^CreERT2/+^* (C/+)*, Nrf2^fl/fl^-Cx3cr1^CreERT2/+^* (cKD), and *Nrf2^-/-^* (tKO) mice were subjected to MCAO. **(A, B)** At 5 h post-reperfusion, mice were sacrificed, and the contralateral (Contra.; C) and ipsilateral (Ipsi.; I) hemispheres of male and female MCAO mice were harvested, followed by mononuclear cell isolation. The isolated cells were stained with antibodies against CD45 and CD11b followed by intranuclear staining with Nrf2 antibody. The cells were then subjected to flow cytometry analysis to assess CD45^int^CD11b^+^ MG positive for Nrf2 expression (n=5/group). **(C, D)** On day 1 post-injury, the mononuclear cells isolated from the contralateral and ipsilateral hemispheres of male and female MCAO mice were stained with CD45 and CD11b antibodies followed by intracellular staining with HO-1 antibody. The cells were then subjected to HO-1 expression assessment in CD45^int^CD11b^+^ MG (n=8/group). **p<0.01; ***p<0.001; N.S.: no significant difference by two-way ANOVA.

To determine whether MG-specific Nrf2 knockdown resulted in attenuated HO-1 expression in the ischemic brain that subsequently led to exacerbated ischemic brain injury after stroke, we assessed the frequency of Nrf2 and HO-1 expression in MG in male and female *Nrf2^fl/fl^-Cx3cr1^CreERT2/^* ^+^ and control *Cx3cr1^CreERT2/^* ^+^ MCAO mice. The frequency of Nrf2 and HO-1 expression in MG was also determined in male and female *Nrf2^-/-^* MCAO mice as additional controls. Our results showed that ischemia induced Nrf2 expression in MG in the ipsilateral hemisphere but not in the contralateral hemisphere of male and female *Cx3cr1^CreERT2/^* ^+^ MCAO mice (**Figures 4A, B**). In contrast, ischemia-induced Nrf2 expression in MG was abolished in the ipsilateral hemisphere of male and female *Nrf2^fl/fl^-Cx3cr1^CreERT2/^* ^+^ and *Nrf2^-/-^* MCAO mice (**Figures 4A, B**). We then assessed whether abolished Nrf2 expression led to attenuated HO-1 expression in *Nrf2^fl/fl^-Cx3cr1^CreERT2/^* ^+^ and *Nrf2^-/-^* MCAO mice. The frequency of HO-1 expression in MG was measured in the ischemic brains of *Cx3cr1^CreERT2/^* ^+^, *Nrf2^fl/fl^-Cx3cr1^CreERT2/^* ^+^, and *Nrf2^-/-^* MCAO mice. Our results showed that ischemic stroke largely increased the frequency of HO-1-expressing MG in the ipsilateral hemisphere in both male and female control *Cx3cr1^CreERT2/^* ^+^ MCAO mice. In contrast, the frequency of HO-1-expressing MG was significantly decreased in the ipsilateral hemisphere of *Nrf2^fl/fl^-Cx3cr1^CreERT2/^* ^+^ MCAO mice compared to that of *Cx3cr1^CreERT2/^* ^+^ MCAO mice in both males and females (**Figures 4C, D**). Notably, a comparable level of HO-1-expressing MG was observed in the ischemic brain of *Nrf2^fl/fl^-Cx3cr1^CreERT2/^* ^+^ and *Nrf2^-/-^* MCAO mice in both males and females (**Figures 4C, D**). Taken altogether, our results demonstrate that ischemia-induced endogenous Nrf2 expression in MG is essential for optimal HO-1 expression in the ischemic brain after stroke.

### MG-specific Nrf2 knockdown modulates inflammatory and anti-inflammatory phenotypes of MG following ischemic stroke

**Figure 5:**
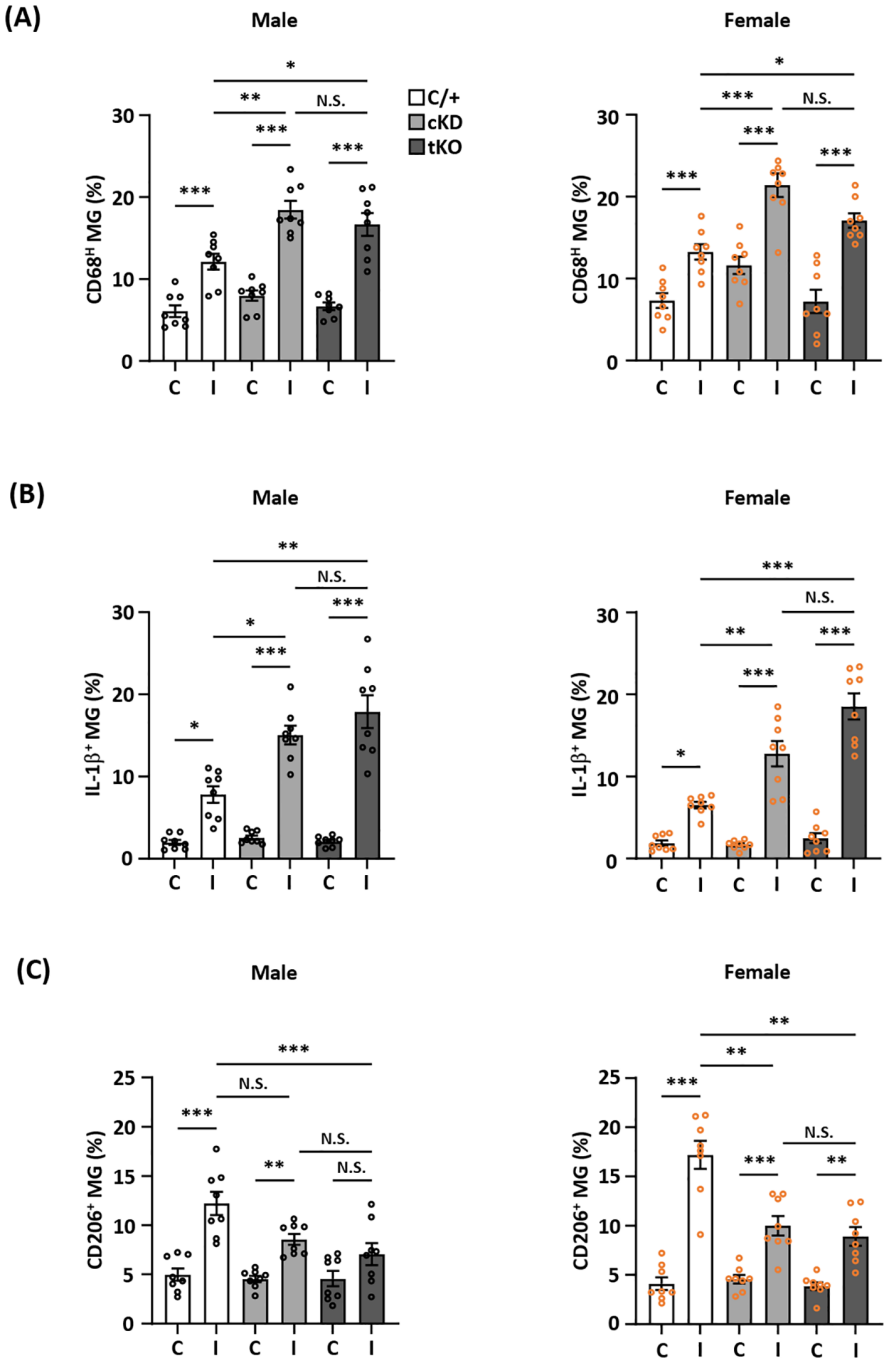
MG-specific Nrf2 knockdown modulates inflammatory and anti-inflammatory phenotypes of MG after ischemic stroke. Male and female *Cx3cr1^CreERT2/+^* (C/+), *Nrf2^fl/fl^-Cx3cr1^CreERT2/+^* (cKD), and *Nrf2^-/-^* (tKO) mice were subjected to MCAO. **(A)** On day 1 post-injury, the contralateral (C) and ipsilateral (I) hemispheres of MCAO mice were harvested, followed by mononuclear cell isolation. The isolated cells were stained with CD45 and CD11b antibodies followed by intracellular staining with CD68 antibody to determine CD45^int^CD11b^+^ MG positive for CD68 expression (n=8/group). **(B)** The isolated cells were ex vivo cultured in the presence of Golgi Plug for 4.5 h followed by surface staining with CD45 and CD11b antibodies. Following fixation and permeabilization, cells were stained with IL-1β antibody to assess the intracellular IL-1β expression in CD45^int^CD11b^+^ MG by flow cytometry (n=8/group). **(C)** On day 2 post-injury, the isolated cells were stained with CD45 and CD11b antibodies followed by intracellular staining with CD206 antibody to assess CD206-expressing CD45^int^CD11b^+^ MG (n=8/group). The representative flow cytometry figures are shown in **Supplementary Figure 2**. *p<0.05; **p<0.01; ***p<0.001; N.S.: no significant difference by two-way ANOVA.

To determine whether Nrf2 knockdown-mediated HO-1 attenuation modulated MG phenotypes in the ischemic brain, we assessed the frequency of CD68/IL-1β- and CD206-expressing MG to assess the inflammatory and anti-inflammatory phenotypes of MG, respectively, in male and female *Nrf2^fl/fl^-Cx3cr1^CreERT2/^* ^+^ and *Cx3cr1^CreERT2/^* ^+^ MCAO mice. *Nrf2^-/-^* MCAO mice were also employed as an additional control. Our results showed that MG-specific Nrf2 knockdown increased the frequency of CD68- and IL-1β-expressing MG but decreased the frequency of CD206-expressing MG in male *Nrf2^fl/fl^-Cx3cr1^CreERT2/^* ^+^ MCAO mice compared to male *Cx3cr1^CreERT2/^* ^+^ MCAO mice (**Figures 5A-C** left; **Supplementary Figure 2** left). Notably, we observed a comparable frequency of CD68-, IL-1β-, and CD206-expressing MG between male *Nrf2^fl/fl^-Cx3cr1^CreERT2/^* ^+^ and *Nrf2^-/-^* MCAO mice (**Figures 5A-C** left; **Supplementary Figure 2** left). Consistently, an increased frequency of CD68- and IL-1β-expressing MG but a decreased frequency of CD206-expressing MG were observed in female *Nrf2^fl/fl^-Cx3cr1^CreERT2/^* ^+^ MCAO mice compared to *Cx3cr1^CreERT2/^* ^+^ MCAO mice, and the frequency of CD68-, IL-1β-, and CD206-expressing MG was comparable between female *Nrf2^fl/fl^-Cx3cr1^CreERT2/^* ^+^ and *Nrf2^-/-^* MCAO mice (**Figures 5A-C** right; **Supplementary Figure 2** right). Taken altogether, these results suggest that ischemia-induced endogenous Nrf2/HO-1 axis activation in MG exerts an essential role in modulating MG phenotypes by promoting anti-inflammatory but suppressing inflammatory phenotypes of MG in the ischemic brain after stroke.

### MG-specific Nrf2 knockdown aggravates BBB disruption after ischemic stroke

**Figure 6:**
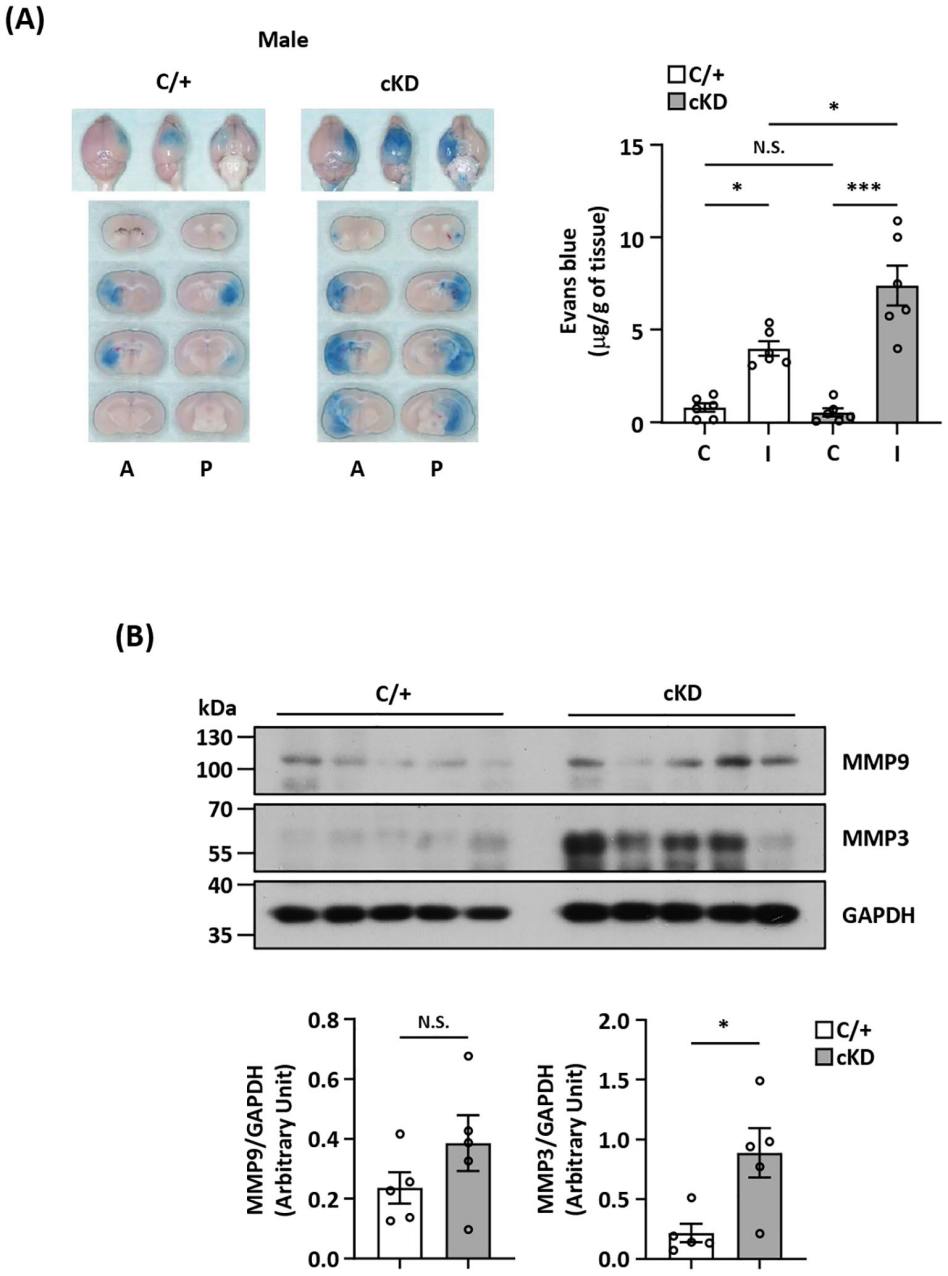
MG-specific Nrf2 knockdown aggravates BBB disruption after ischemic stroke. **(A)** *Cx3cr1^CreERT2/+^* (C/+) and *Nrf2^fl/fl^-Cx3cr1^CreERT2/+^ (*cKD*)* mice were subjected to 3 h MCAO followed by 3.5 h reperfusion. One hour prior to sacrifice, mice were i.v. injected Evans blue. At 6.5 h post-injury, the ischemic brains were harvested and subjected to sectioning and imaging, and the Evans blue leakage in the contralateral and ipsilateral hemispheres was quantified (n=6/group). Representative ischemic brain images of C/+ and cKD MCAO mice displaying Evans blue extravasation are shown, and the amount of Evans blue extravasation of the contralateral (C) and ipsilateral (I) hemispheres was also quantified. A, anterior surface; P, posterior surface. *p<0.05; ***p<0.001; N.S.: no significant difference by two-way ANOVA. **(B)** The ipsilateral hemispheres harvested from C/+ and cKD MCAO mice were subjected to western blot analysis for MMP9 and MMP3 expression. The levels of MMP9 and MMP3 expression were also quantified (n=5/group). *p<0.05; N.S.: no significant difference by unpaired *t* test.

MG play an active role in maintaining BBB stabilization (40, 41). To investigate whether ischemia-induced endogenous Nrf2/HO-1 axis activation in MG exerted a protective effect in preventing excessive BBB disruption in the ischemic brain after stroke, we assessed the level of BBB disruption in the ischemic brains of *Cx3cr1^CreERT2/+^* and *Nrf2^fl/fl^-Cx3cr1^CreERT2/+^* MCAO mice. We observed an augmented Evans blue leakage in the ipsilateral hemisphere of *Nrf2^fl/fl^-Cx3cr1^CreERT2/+^* MCAO mice compared to that of control *Cx3cr1^CreERT2/+^* MCAO mice, indicating MG-specific Nrf2 knockdown aggravates BBB disruption after stroke (**Figure 6A**). Notably, there was no increased Evans blue leakage in the contralateral hemisphere when comparing *Nrf2^fl/fl^-Cx3cr1^CreERT2/+^* with *Cx3cr1^CreERT2/+^* MCAO mice, suggesting MG-specific Nrf2 knockdown doesn’t alter the integrity of BBB under no injury condition (**Figure 6A**). Since the induction of MMP3 and MMP9 has been shown to promote BBB disruption (42, 43), we analyzed the levels of MMP3 and MMP9 expression in the ischemic brains of *Cx3cr1^CreERT2/+^* and *Nrf2^fl/fl^-Cx3cr1^CreERT2/+^* MCAO mice. Our results showed that MMP3 and MMP9 expression in the ischemic brains were elevated in *Nrf2^fl/fl^-Cx3cr1^CreERT2/+^* MCAO mice compared to control *Cx3cr1^CreERT2/+^* MCAO mice (**Figure 6B**; **Supplementary Figure 3**). Collectively, these findings demonstrate that MG-specific Nrf2 knockdown leads to augmented MMP3 and MMP9 expression in the ischemic brain, and that may subsequently result in aggravated BBB disruption after stroke.

### Diabetic stroke exhibits attenuated endogenous Nrf2/HO-1 axis activation in MG and exacerbated ischemic brain injury

**Figure 7:**
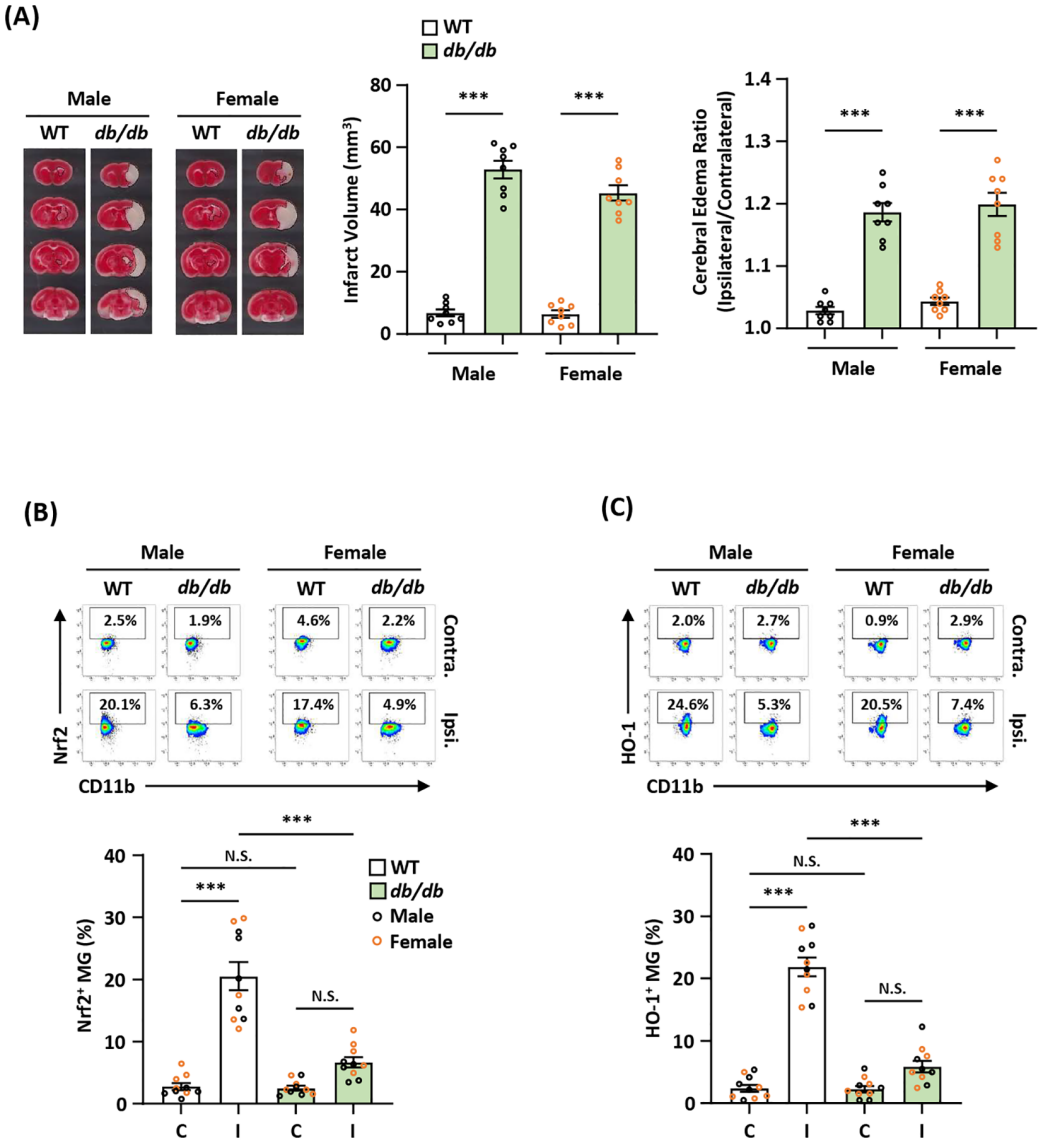
Diabetic stroke exhibits attenuated Nrf2/HO-1 expression in MG and exacerbated ischemic brain injury. **(A)** Male and female WT and *Lepr^db/db^* (*db/db*) mice were subjected to MCAO and sacrificed at 5 h post-reperfusion. The ischemic brains were harvested and subjected to TTC staining. One representative TTC-stained brain sample from each group is shown, and the infarct volumes were measured. The edema ratio was also calculated (n=8/group). ***p<0.001 by unpaired *t* test. **(B, C)** The ischemic brains harvested from male and female WT and *db/db* MCAO mice were subjected to mononuclear cell isolation. The isolated cells were then stained with CD45 and CD11b antibodies followed by intranuclear staining or intracellular staining with Nrf2 or HO-1 antibodies to determine CD45^int^CD11b^+^ MG positive for **(B)** Nrf2 or **(C)** HO-1 expression, respectively (n=5/sex/group). ***p<0.001; N.S.: no significant difference by two-way ANOVA.

Diabetes is a well-established risk factor for ischemic stroke and has been shown to worsen ischemic brain injury after stroke (44). Notably, attenuated Nrf2 and HO-1 expression was found in diabetes (45–47). To elucidate whether exacerbated ischemic brain injury observed in diabetic stroke is associated with attenuated endogenous Nrf2/HO-1 axis activation in MG, male and female type II diabetic *Lepr^db/db^* mice were subjected to MCAO to assess the severity of ischemic brain injury. Consistent with previous findings (48–50), we observed that male and female *Lepr^db/db^* MCAO mice displayed significantly enlarged infarct volumes and elevated cerebral edema compared to control WT MCAO mice (**Figure 7A**). We then analyzed the frequency of Nrf2-expressing MG in the ischemic brain of *Lepr^db/db^* and WT MCAO mice. Our results showed that the frequency of Nrf2-expressing MG was significantly decreased in the ipsilateral hemisphere of *Lepr^db/db^* MCAO mice compared to that of WT MCAO mice in both males and females (**Figure 7B**). Furthermore, we observed that the frequency of HO-1-expressing MG was largely reduced in the ipsilateral hemisphere of *Lepr^db/db^* MCAO mice compared to that of WT MCAO mice in both males and females (**Figure 7C**). Collectively, our results demonstrate that diabetes exacerbates ischemic brain injury after stroke, and that is associated with attenuated endogenous Nrf2/HO-1 axis activation in MG in diabetic stroke.

### Pharmacological activation of exogenous Nrf2/HO-1 axis in MG ameliorates diabetes-exacerbated ischemic brain injury after stroke

**Figure 8:**
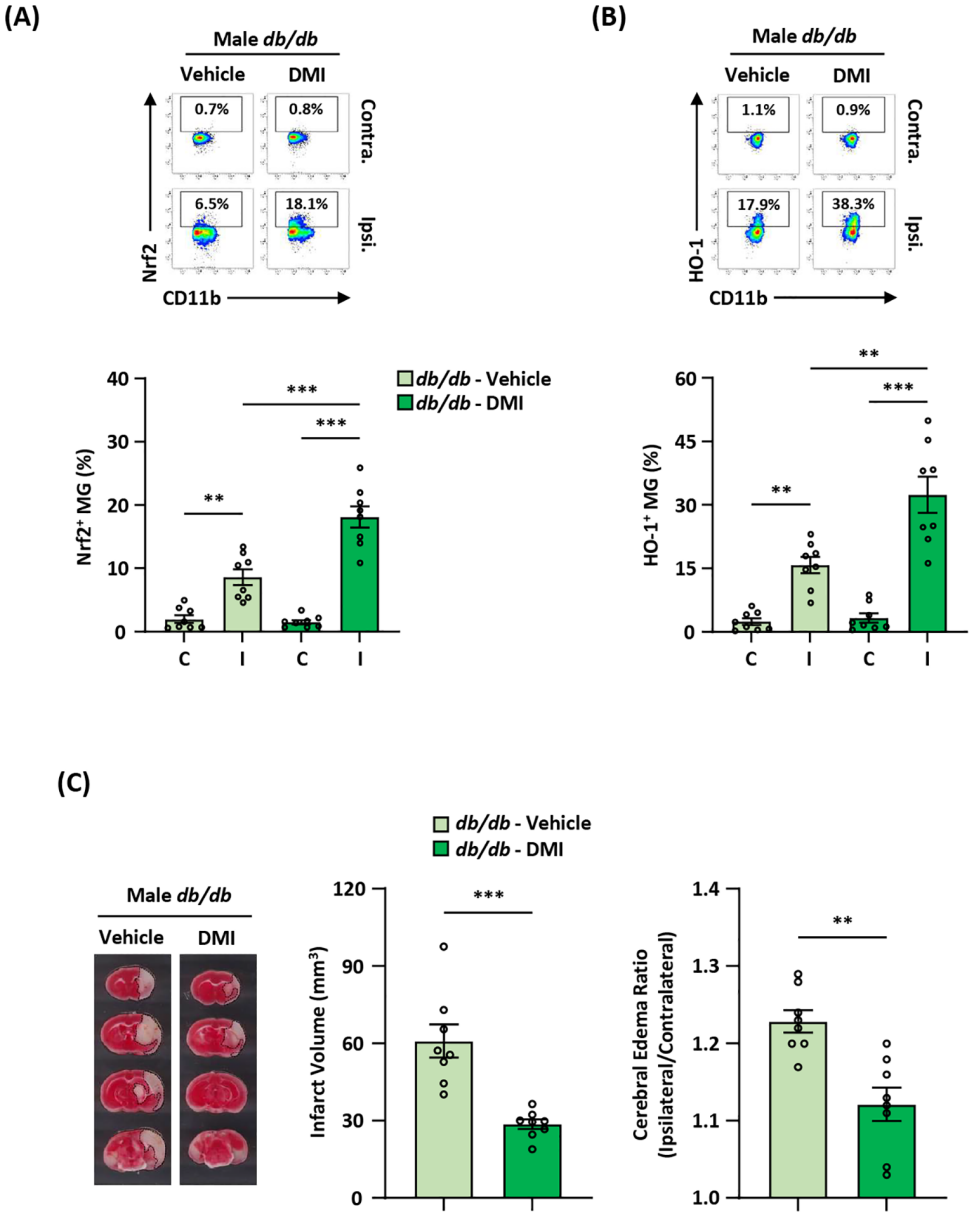
Pharmacological activation of exogenous Nrf2/HO-1 axis in MG ameliorates diabetes-exacerbated ischemic brain injury. Male *Lepr^db/db^* (*db/db*) mice were subjected to MCAO and then treated with vehicle (n=8) or DMI (n=8) at 1 h post-reperfusion. At 15 h post-injury, the ischemic brains were harvested, followed by mononuclear cell isolation. **(A, B)** The isolated cells were then stained with antibodies against CD45 and CD11b in the presence of Nrf2 or HO-1 antibodies to assess CD45^int^CD11b^+^ MG positive for **(A)** Nrf2 or **(B)** HO-1 expression, respectively. **p<0.01; ***p<0.001 by two-way ANOVA. **(C)** The ischemic brains were harvested from vehicle- and DMI-treated *db/db* MCAO mice at 15 h post-injury and subjected to TTC staining (n=8/group). One representative TTC-stained brain sample from vehicle- and DMI-treated *db/db* MCAO mice is shown. The infarct volumes were measured, and the edema ratio was also calculated (n=8/group). **p<0.01; ***p<0.001 by unpaired *t* test.

To elucidate whether the induction of exogenous Nrf2/HO-1 axis activation in MG ameliorated ischemic brain injury in diabetic stroke, *Lepr^db/db^* MCAO mice were administered DMI, and the ischemic brains were harvested to assess Nrf2 and HO-1 expression in MG. DMI, a cell-permeable itaconate derivative, is capable of inducing Nrf2 protein level and that subsequently induces downstream target genes, including HO-1 and glutathione (27, 33, 51, 52). Our results showed that the frequency of Nrf2-expressing MG was significantly increased in the ipsilateral hemisphere of DMI-treated *Lepr^db/db^* MCAO mice compared to that of vehicle-treated *Lepr^db/db^* MCAO mice (**Figure 8A**). Furthermore, we observed that the frequency of HO-1-expressing MG was largely elevated in the ipsilateral hemisphere of DMI-treated *Lepr^db/db^* MCAO mice compared to that of vehicle-treated *Lepr^db/db^* MCAO mice (**Figure 8B**). Most importantly, when comparing the severity of ischemic brain injury in vehicle- and DMI-treated *Lepr^db/db^* MCAO mice, we found that DMI-treated *Lepr^db/db^* MCAO mice exhibited ameliorated brain injury with attenuated infarct volumes and lessened cerebral edema compared to vehicle-treated *Lepr^db/db^* MCAO mice (**Figure 8C**). Altogether, our results demonstrate that the induction of exogenous Nrf2/HO-1 axis activation in MG ameliorates diabetes-exacerbated ischemic brain injury after stroke.

## Discussion

The induction of Nrf2/HO-1 axis activation has been shown to offer protection against CNS diseases, including ischemic stroke, Parkinson’s disease, and Multiple Sclerosis (19, 21, 32, 34). Using Nrf2 activators, studies have demonstrated that the activation of exogenous Nrf2/HO-1 axis exerts anti-inflammatory and antioxidant effects in ischemic stroke (13). In this study, we demonstrated that ischemic stroke induced endogenous Nrf2 expression in MG that subsequently promoted HO-1 expression in MG in the ischemic brain. Notably, using MG-specific Nrf2 knockdown mice we showed that ischemia-induced endogenous Nrf2/HO-1 axis activation in MG was largely suppressed in the ischemic brain. Importantly, MG-specific Nrf2 knockdown led to exacerbated ischemic brain injury, suggesting the protective effect of endogenous Nrf2/HO-1 axis activation in MG following ischemic stroke. At the cellular level, we found that ischemia induced a high frequency of HO-1-expressing MG in the ischemic brain at day 1 and day 2 post-injury, however, the frequency of HO-1-expresing MG was then attenuated at day 3 post-injury. Notably, the increased frequency of HO-1-expressing MG was correlated with an increased frequency of CD206-expressing MG and a decreased frequency of CD68-expressing MG at day 2 post-injury. Conversely, the attenuated frequency of HO-1-expressing MG was linked to a decreased frequency of CD206-expressing MG and an increased frequency of CD68-expressing MG at day 3 post-injury. CD206 is a marker for border-associate macrophages (BAM) and an M2 marker for macrophages (53, 54). In addition, studies have shown that MG with anti-inflammatory phenotypes also express CD206 (26, 55, 56). Although both microglia and BAM express CD45, they express different levels of CD45, where BAM express a high level of CD45, and MG express an intermediate level of CD45 (57). In our results, we observed a low frequency of CD206^+^ MG at day 1 post-injury and a significantly increased frequency of CD206^+^ MG at day 2 post-injury. The increased CD206^+^ cells expressed an intermediated level of CD45 (**Supplementary Figure 4**), suggesting the potential conversion of CD206^-^ MG into CD206^+^ MG. Collectively, these results suggest that ischemia-induced endogenous Nrf2/HO-1 axis activation in MG may play an essential role in modulating MG phenotypes through regulating CD206 and CD68 expression in MG after stroke.

We observed an early induction of anti-inflammatory MG phenotype followed by a late induction of persistent inflammatory MG phenotype in the ischemic brain, which is consistent with previous findings (58). However, there is a scientific gap with regard to what pathway is involved in modulating anti-inflammatory and inflammatory MG phenotypes after ischemic stroke. Here, we show that the induction of early anti-inflammatory MG phenotype in the ischemic brain is driven by ischemia-induced endogenous Nrf2/HO-1 axis activation in MG. Subsequently, the induction of late inflammatory MG phenotype in the ischemic brain is associated with attenuated endogenous Nrf2/HO-1 axis activation in MG. These interplays are demonstrated by utilizing MG-specific Nrf2 knockdown mice, as we found that the ablation of endogenous Nrf2/HO-1 axis activation in MG resulted in attenuated anti-inflammatory CD206-expressing MG and aggravated inflammatory CD68-expressing MG in the ischemic brain. Ultimately, these altered balances led to exacerbated ischemic brain injury in stroke mice with MG-specific Nrf2 knockdown. Thus, our findings revealed previously unestablished protective mechanisms by which ischemia-induced endogenous Nrf2/HO-1 axis activation modulates MG phenotypes that subsequently restrains ischemic brain injury after stroke. Notably, HO-1 possesses anti-inflammatory, antioxidant, and neuroprotective effects (59). Previous studies have demonstrated that HO-1 promotes BDNF expression (60, 61). Mechanistically, the downstream product of HO-1, bilirubin, was shown to activate the PI3K-Akt pathway, resulting in increased BDNF expression in glia-enriched cultures (62). BDNF plays a significant role in the prognosis, pathogenesis, and rehabilitation in stroke (63–65). Studies have shown that a low circulating BDNF level was associated with poor long-term functional outcomes after stroke (66), and positive outcomes have been linked to increased BDNF levels after stroke (65, 67). Thus, ischemia-induced endogenous Nrf2/HO-1 axis could promote BDNF production that subsequently enhances neurogenesis, leading to improved functional outcomes after stroke.

The BBB plays an essential role in maintaining CNS homeostasis. The integrity of BBB is maintained by physical and biochemical characteristics, including tight junction protein complexes and transporters (68, 69). Furthermore, its function is supported by astrocytes, pericytes, neurons, and MG, forming the neurovascular unit (NVU). The cellular components of NVU can be activated upon sterile inflammation or disease induction, which contributes to BBB remodeling. This is particularly true for MG because they can be polarized into inflammatory or anti-inflammatory phenotypes that exert different impacts on BBB integrity. Studies have shown that inflammatory MG promote BBB dysfunction, whereas anti-inflammatory MG contribute to maintaining BBB integrity. We observed that MG-specific Nrf2 knockdown stroke mice that exerted attenuated anti-inflammatory and aggravated pro-inflammatory MG phenotypes displayed exacerbated BBB disruption in the ischemic brain. In addition, we found that MMP3 and MMP9, which have been shown to play an essential role in promoting BBB disruption (42, 43, 70), were upregulated in the ischemic brains of MG-specific Nrf2 knockdown stroke mice compared to those of control stroke mice. Since tight junction proteins, including Claudin-5, Occludin, and ZO-1, play an important role in maintaining homeostasis of BBB (71), elevated MG activation and enhanced MMP3/9 production in MG-specific Nrf2 knockdown stroke mice may potentially disrupt these proteins in cerebral vessels leading to BBB breakdown. Collectively, our results demonstrate that ischemia-induced endogenous Nrf2/HO-1 axis activation in MG contributes to maintaining BBB integrity in the ischemic brain through modulating MG phenotypes.

Diabetes is a well-established risk factor for stroke and has been shown to worsen brain injury after ischemic stroke. Using the type II diabetic mouse model, we observed that *Lepr^db/db^* mice subjected to MCAO developed severe brain injury with enlarged infarct volumes and elevated cerebral edema compared to WT MCAO mice. Furthermore, attenuated endogenous Nrf2/HO-1 axis activation in MG was observed in *Lepr^db/db^* MCAO mice compared to WT MCAO mice. Importantly, the treatment of *Lepr^db/db^* MCAO mice with DMI, which has been shown to induce Nrf2/HO-1 axis activation by our group and others (33, 69), led to the induction of exogenous Nrf2/HO-1 axis in MG and ameliorated ischemic brain injury. Previous studies demonstrate that elevated neuroinflammation and aggravated BBB disruption contribute to exacerbated ischemic brain injury in diabetes after stroke (72, 73). As our results show that diabetic stroke mice phenocopy MG-specific knockdown stroke, our findings showing that Nrf2/HO-1 axis attenuation led to elevated inflammatory MG and aggravated BBB disruption in the ischemic brain provide additional cellular and molecular mechanisms by which diabetes exacerbates brain injury in ischemic stroke. Additionally, DMI, a derivative of itaconate that can inhibit succinate dehydrogenase (SDH), could potentially modulate ischemic brain injury through SDH/succinate axis. During respiration with normal oxygen consumption, SDH catalyzes succinate to fumarate in the tricarboxylic acid cycle (74). During ischemia, SDH works reversely that results in succinate accumulation. Previously studies have shown that accumulated succinate was detected in the ischemic tissues including heart, kidney, liver, and brain (75). Subsequently, accumulated succinate is rapidly oxidized following reperfusion, leading to increased reactive oxygen species production. Importantly, the inhibition of SDH activity has been shown to confer protection against ischemic injury (75, 76). Thus, DMI could potentially modulate ischemic brain injury via Nrf2-dependent (Nr2/HO-1 axis) and Nrf2-independent (SDH/succinate axis) pathways. Finally, although tissue plasminogen activator (tPA) is an FDA-approved drug for acute ischemic stroke treatment, diabetes increases the risk of hemorrhagic transformation after tPA therapy (77). Importantly, Nrf2 activation has been shown to ameliorate hemorrhagic transformation and alleviate BBB injury in focal cerebral ischemia (78, 79). Thus, it would be beneficial to consider the combinational therapy with tPA and DMI for diabetic stroke treatment because it would allow tPA to reestablish cerebral blood perfusion and DMI to attenuate diabetes-exacerbated cerebral infarct and hemorrhagic transformation in the ischemic brain.

There are limitations to our current study. First, we define inflammatory and anti-inflammatory MG in the ischemic brain based on their expression of CD68 and CD206, respectively. In the future study, analyzing additional markers for inflammatory MG, such as CD16 and IL-1α, and anti-inflammatory MG, such as Arg1 and YM1, would further strengthen the essential role of endogenous Nrf2/HO-1 axis activation in modulating MG polarization after stroke. Second, Nrf2 is highly expressed in microglia and brain endothelial cells in the CNS (80). In this study, we observed MG-specific Nrf2 knockdown MCAO mice exhibit exacerbated ischemic brain injury. In addition, we found that MG-specific Nrf2 knockdown MCAO mice displayed a comparable level of brain injury compared to total Nrf2 knockout MCAO mice. These results suggest that Nrf2-expressing MG play the main role in restraining ischemic brain injury after stroke. However, it may be beneficial to elucidate whether ischemia-induced endogenous Nrf2 expression in brain endothelial cells plays a role in maintaining the integrity of BBB after stroke. Third, the induction of Nrf2 activation leads to the production of a variety of phase II antioxidant genes, including HO-1, GCLC, and NQO1. In this study, we focused on analyzing HO-1 expression in MG after stroke. Thus, it would be worth assessing whether NQO1 and GCLC are induced in MG after ischemic stroke and determining their effects on modulating ischemic brain injury. Fourth, because MG-specific Nrf2 knockdown stroke mice exhibited more than 60% and 70% mortality by day 4 and day 6 post-injury, it limits us to conduct animal behavior tests such as rotarod tests to assess their motor function. However, with elevated cerebral infarct volume and exacerbated BBB leakage in MG-specific Nrf2 knockdown stroke mice, we believe that they would have a poor outcome of rotarod tests compared to control stroke mice. Finally, aging is the major risk factor for cardiovascular disease, and it has been shown that Nrf2 activation is impaired in aging (81, 82). In this study, only young but not aged animals were used. Thus, future study using aged animals to assess whether ischemia-induced endogenous Nrf2/HO-1 activation in MG can also be observed to restrain ischemic brain injury would further strengthen the protective effect of endogenous Nrf2/HO-1 axis activation in MG after ischemic stroke.

In this study, we identify ischemia induces endogenous Nrf2/HO-1 axis activation in MG after stroke. The activation of Nrf2/HO-1 axis subsequently promotes the induction of anti-inflammatory MG, resulting in restraining ischemic brain injury after stroke. Furthermore, we demonstrate that MG-specific Nrf2 knockdown results in attenuated endogenous Nrf2/HO-1 axis activation, leading to suppressed CD206 expression and enhanced CD68 expression in MG, and that subsequently aggravates neuroinflammation and exacerbates brain injury in ischemic stroke. In addition, our results show that attenuated endogenous Nrf2/HO-1 axis activation in MG is responsible for exacerbated ischemic brain injury in diabetes with stroke. Notably, the pharmacologic activation of exogenous Nrf2/HO-1 axis in MG can ameliorate exacerbated ischemic brain injury in diabetic stroke. In summary, our study provides insights into how ischemia-induced endogenous Nrf2/HO-1 axis activation modulates MG phenotypes and restrains ischemic brain injury and reveals that diabetes-induced attenuation of endogenous Nrf2/HO-1 axis activation in MG exacerbates ischemic brain injury after stroke. Thus, our findings strengthen the therapeutic potential of targeting Nrf2/HO-1 axis activation in MG for the treatment of ischemic stroke and offer translational potential of clinical interventions for diabetic stroke.

## Data Availability

The original contributions presented in the study are included in the article/**Supplementary Material**. Further inquiries can be directed to the corresponding author.
