# Supplementary material for: Ischemia-induced endogenous Nrf2/HO-1 axis activation modulates microglial polarization and restrains ischemic brain injury
Source: Front Immunol. 2024 Oct 14;15:1440592. doi: 10.3389/fimmu.2024.1440592 (PMC11513276; doi:10.3389/fimmu.2024.1440592)
Supplement: Supplementary file 1 [file DataSheet1.pdf]

Supplementary Fig. 1

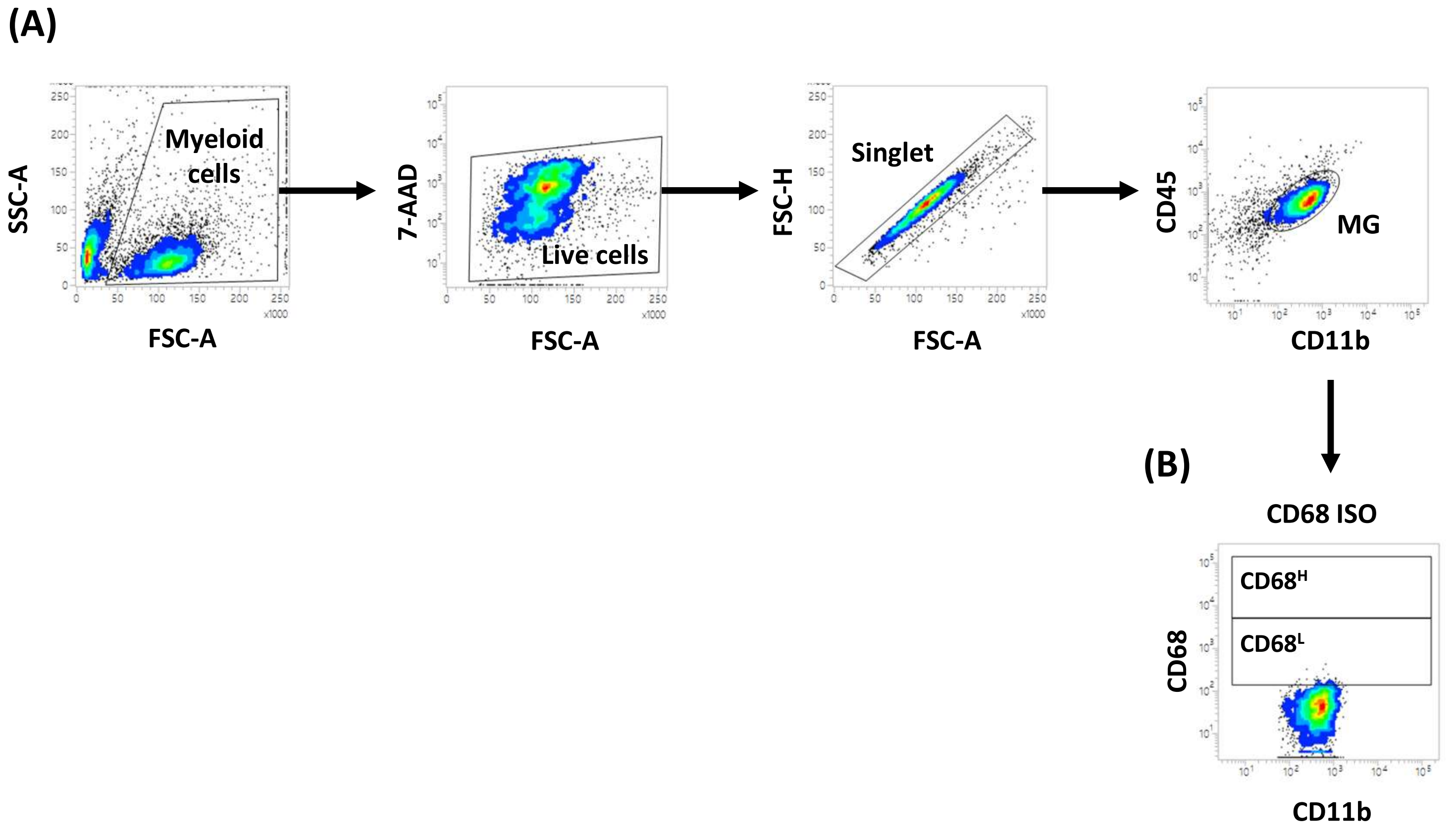

Supplementary Figure 1 Gating strategy of flow cytometry analysis. (A) Mononuclear cells isolated from the contralateral hemisphere of ischemic brain were subjected to surface staining of CD11b and CD45 antibodies in the presence of 7-AAD. 7-AAD negative was presented as live cells, the single cell population was gated by singlet gating. MG were gated based on the intermediate expression of CD45 ($CD45^{int}$) and positive expression of CD11b ($CD11b^{+}$). **(B)** For the CD68 staining, CD68 low MG population ($CD68^{L}$) was gated based on the CD68 isotype.

Supplementary Fig. 2

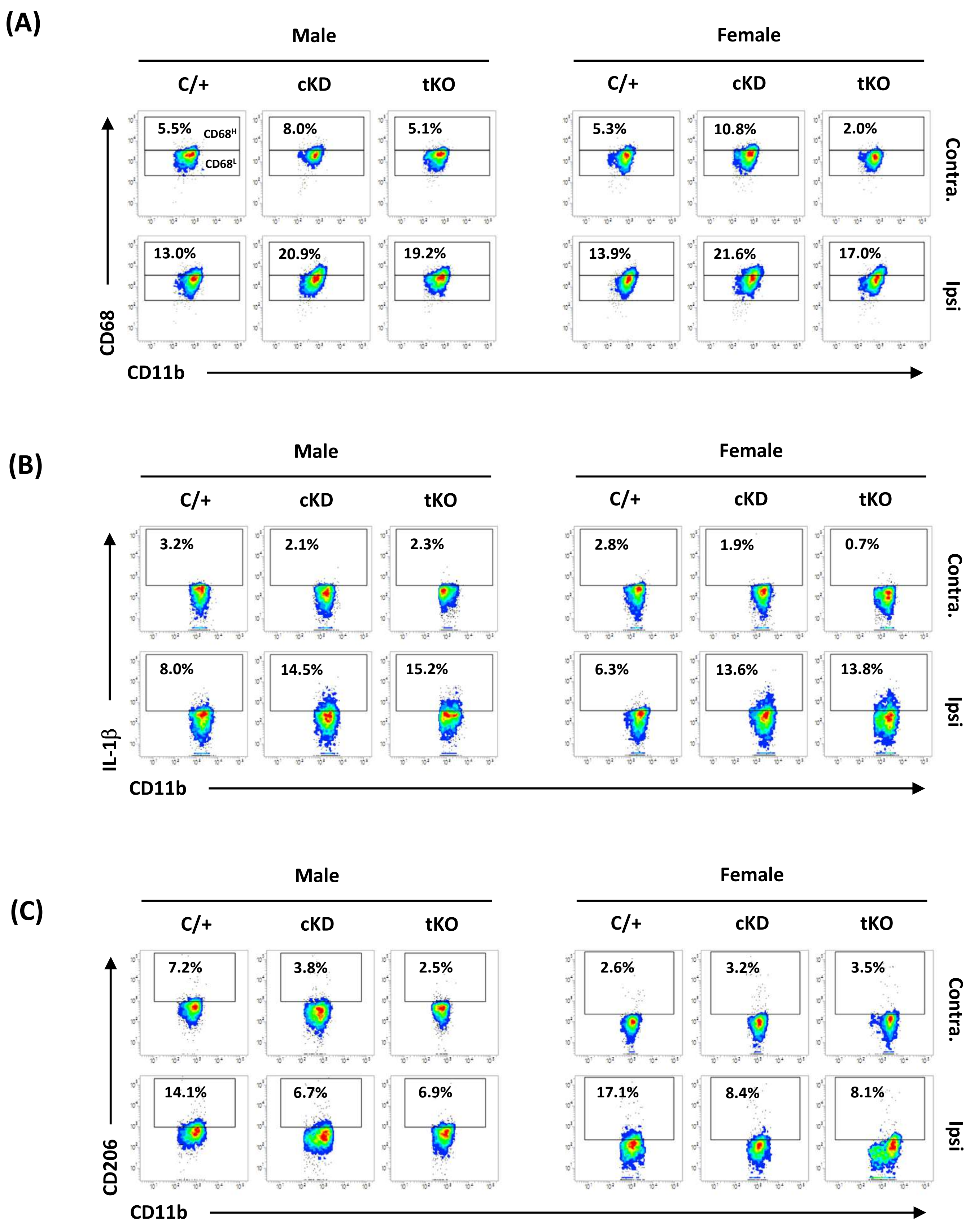

Supplementary Figure 2 The representative flow cytometry figures of Figure 5.

Supplementary Fig. 3

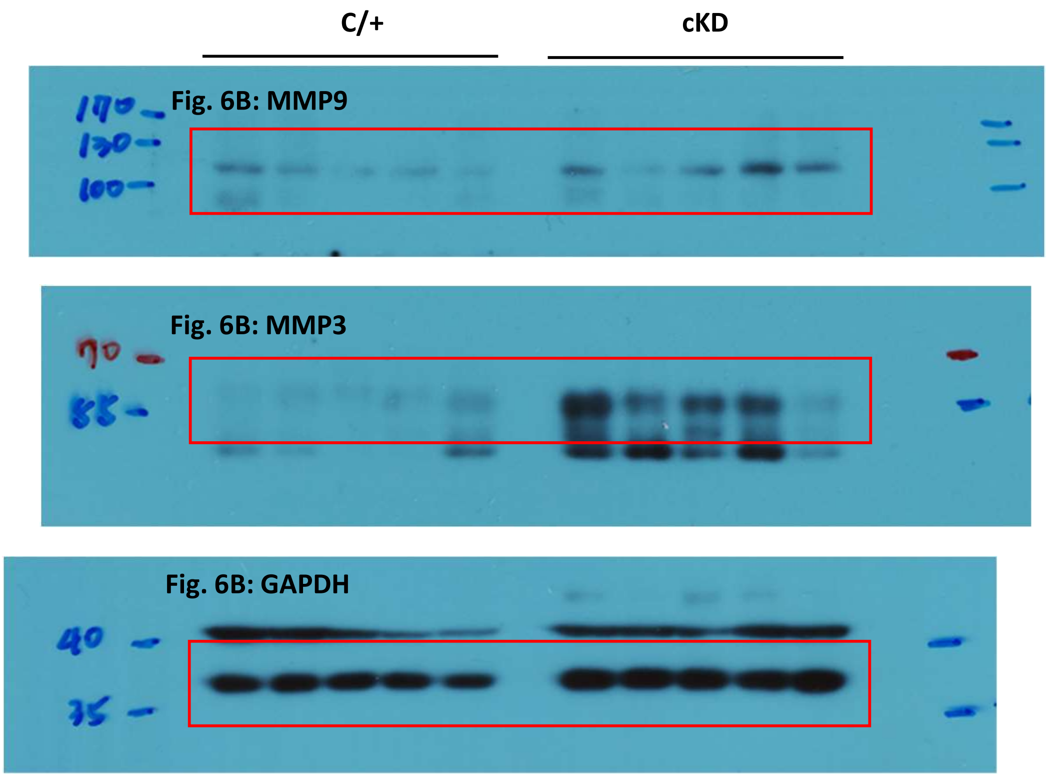

Supplementary Figure 3 Original western blot images. The original non-cropped X-ray film images of Fig. 6B.

Supplementary Fig. 4

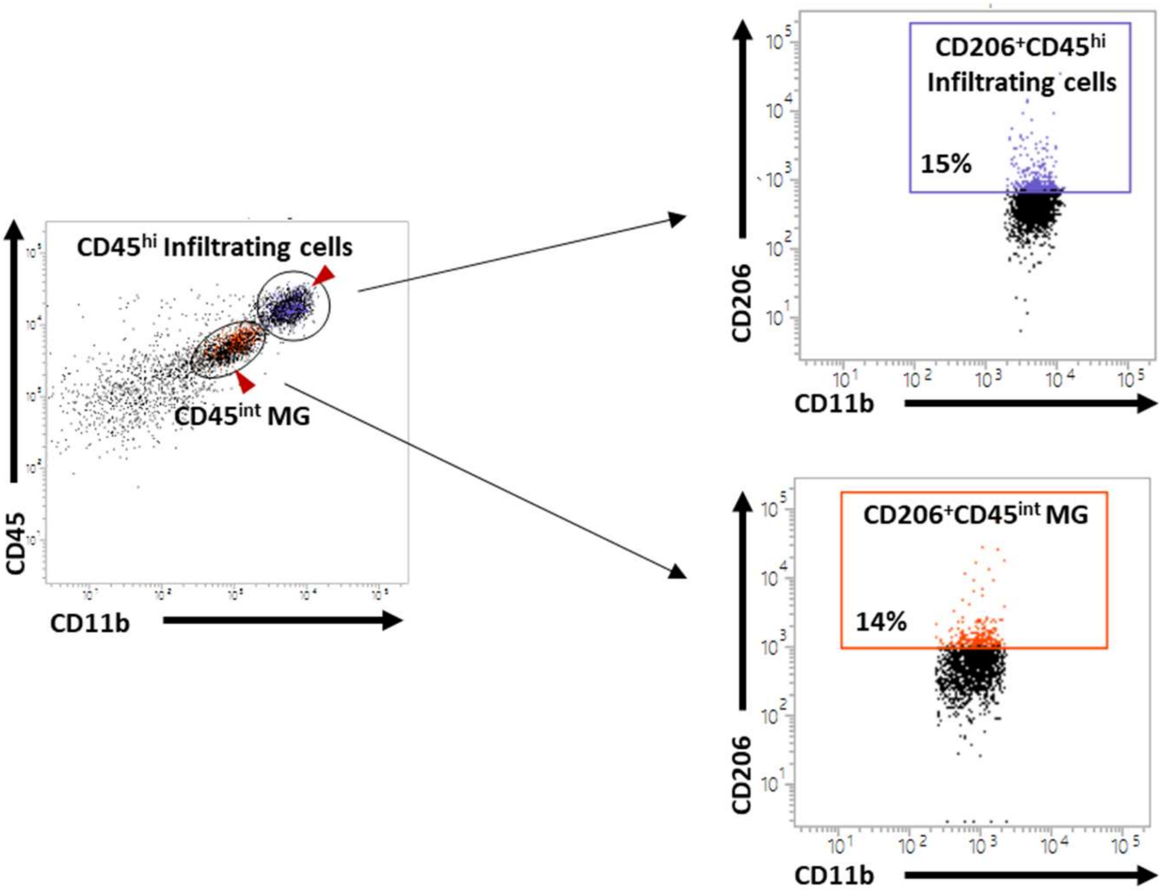

Supplementary Figure 4 CD206^{+} MG exhibit intermediate expression of CD45. Mononuclear cells isolated from the ipsilateral hemisphere of MCAO mice at day 2 post-injury were subjected to surface staining of CD11b and CD45 antibodies followed by fixation and permeabilization. Cells were then stained with CD206 followed by flow cytometry analysis. Red dots represent CD206^{+} CD45^{int}CD11b^{+} MG. Purple dots represent CD206^{+} CD45^{hi}CD11b^{+} infiltrating cells.
